# Using Technology for Prescription and Adherence in an Alzheimer’s Prevention Program

**DOI:** 10.1093/geroni/igab046.1674

**Published:** 2021-12-17

**Authors:** Amber Watts, Angela VanSciver, Jon Clutton, Katrina Finley, Erica Flores, Amanda Szabo-Reed, Jeffrey Burns, James Vacek

**Affiliations:** 1 University of Kansas, Lawrence, Kansas, United States; 2 University of Kansas Medical Center, Fairway, Kansas, United States; 3 University of Kansas Medical Center, Kansas City, Kansas, United States

## Abstract

Healthy lifestyle change is difficult to adopt and maintain without support. Often physicians recommend exercise to their patients, but have limited means to support this change. A major goal of our study is to provide physicians with a simple method of referring patients to a program that supports adoption and maintenance of exercise that meets recommended guidelines for older adults. The Lifestyle Empowerment for Alzheimer’s Prevention program (LEAP! Rx) is a yearlong intervention to support cognitively normal older adults in adoption and maintenance of moderate to vigorous exercise, a key prevention factor for Alzheimer’s disease. The program uses the electronic medical record and builds relationships with physicians to identify patients eligible to participate. It electronically communicates about patients’ progress back to referring physicians to facilitate ongoing physician-patient interaction. Participants receive exercise coaching to reach their weekly exercise goals and have access to online lifestyle education classes (e.g., nutrition, sleep, stress management). The study is currently enrolling (n= 121 enrolled; mean age 71.4; 12% non-white, 4% Hispanic/Latino, and 83% female). Physician referrals originate from five clinics represented by 48 physicians. The study design will actively compare the physician referral process to self-referrals from the community (n=20). We have adapted the protocol to the conditions of the pandemic including online exercise coaching and support. This presentation will discuss successes and lessons learned from this novel method of recruitment and adherence to exercise.

